# Biomarker-defined congestion profiles in very old adults with acute heart failure: beyond ejection fraction classification

**DOI:** 10.1007/s41999-026-01505-y

**Published:** 2026-05-25

**Authors:** Pau Llàcer, François Croset, Alberto Pérez Nieva, Jorge Campos, Marina García Melero, Carlos Pérez, Marina Vergara, Paul Cevallos Castro, Juanes Oyuela Rodriguez, Cristina Fernández, María Pumares, Martín Fabregate, Beatriz Del Hoyo, Esteban Pérez Pisón, Luis Manzano

**Affiliations:** 1 Internal Medicine Department, Hospital Universitario Ramón y Cajal, IRYCIS, Madrid, Spain; 2 Department of Medicine and Medical Specialties, Facultad de Medicina y Ciencias de La Salud, Universidad de Alcalá, IRYCIS, Madrid, Spain

**Keywords:** Acute heart failure, Congestion, Biomarkers, BNP, CA125, Profiles, Older adults, Ejection fraction

## Abstract

**Background:**

Congestion is the main driver of worsening acute heart failure, yet its manifestation in very old adults and its relationship with prognosis remains incompletely understood. Traditional classification based on left ventricular ejection fraction (LVEF) may not adequately capture the biological heterogeneity of congestion in this population. We aimed to characterize patient subgroups defined by BNP and CA125 constellations, to characterize their clinical and echocardiographic features, and to evaluate their prognostic implications. As a secondary objective, we explored their relationship with conventional LVEF categories.

**Methods:**

We conducted a retrospective cohort study including 830 consecutive patients hospitalized with acute heart failure. Congestion was assessed using clinical signs, echocardiography, biomarkers (BNP and CA125), and point-of-care ultrasound. Outcomes included heart failure rehospitalization and all-cause mortality over a median follow-up of 310 days (IQR 62–543). Multivariable Cox regression models were used.

**Results:**

The median age was 87 years, and 65.6% were women. Four patient subgroups were defined by BNP and CA125 constellations, showing distinct clinical and echocardiographic profiles. Mortality increased progressively across phenotypes, with combined elevation of BNP and CA125 independently associated with higher risk of death (HR 1.48; *p* = 0.008). Isolated CA125 elevation was associated with increased rehospitalization risk (HR 1.45; *p* = 0.031). In contrast, LVEF categories showed limited prognostic discrimination.

**Conclusions:**

In very old adults hospitalized with acute heart failure, combined elevation of BNP and CA125 was independently associated with increased mortality, while isolated CA125 elevation was associated with higher rehospitalization risk. LVEF was not independently associated with either outcome. These findings suggest that integrating BNP and CA125 may complement conventional LVEF classification for risk stratification in this population.

**Supplementary Information:**

The online version contains supplementary material available at https://doi.org/10.1007/s41999-026-01505-y.

## Key summary points

**Aim:**

To characterize patient subgroups defined by BNP and CA125 constellations in very old adults hospitalized with acute heart failure, to describe their clinical and echocardiographic correlates, and to evaluate their prognostic implications beyond conventional left ventricular ejection fraction (LVEF) classification.

**Findings:**

Four patient subgroups were defined by BNP and CA125 constellations, showing distinct clinical, echocardiographic, and prognostic profiles. Combined elevation of BNP and CA125 associated with increased mortality, while isolated CA125 elevation was associated with higher rehospitalization risk. In contrast, LVEF categories showed limited prognostic discrimination.

**Message:**

In very old adults hospitalized with acute heart failure, combined elevation of BNP and CA125 was independently associated with increased mortality, while isolated CA125 elevation was associated with higher rehospitalization risk. These findings suggest that integrating BNP and CA125 may complement conventional LVEF classification for risk stratification in this population.

**Supplementary Information:**

The online version contains supplementary material available at https://doi.org/10.1007/s41999-026-01505-y.

## Introduction

Congestion is the main driver of worsening heart failure [1]. Traditionally, congestion in heart failure (HF) has been considered a hemodynamic concept, defined as increased central filling pressures resulting from fluid accumulation in intravascular and extravascular compartments [2, 3]. However, congestion is not synonymous with fluid overload, as elevated intracardiac pressures do not always correlate with total blood volume expansion, and vice versa [2, 3]. Fluid redistribution, for example, represents a form of congestion without fluid overload and is typical in acute pulmonary edema, whereas acutely decompensated HF is more commonly characterized by fluid overload [1].

Optimal characterization of congestion through a multiparametric approach, integrating clinical, imaging, and biomarker data, may improve risk stratification and guide management in patients with acute heart failure.[4]. Although pathophysiological differences between heart failure with reduced ejection fraction (HFrEF) and preserved ejection fraction (HFpEF) are well established in chronic settings [5, 6], these distinctions become less clear during acute episodes. Accordingly, acute congestion management does not differ substantially according to left ventricular ejection fraction (LVEF) [3, 4, 7].

Older patients represent an increasing proportion of acute HF admissions and have worse prognosis [8, 9], yet remain underrepresented in clinical trials [10]. Data on congestion profiles and prognostic determinants in this age group are scarce. Although LVEF-based classification has been central to heart failure taxonomy, its ability to capture the heterogeneity of congestion and to inform prognosis in very old patients with acute heart failure is limited [7, 11, 12]. In this population, clinical presentation is frequently shaped by multimorbidity, frailty, and systemic congestion, which may complicate diagnosis and obscure the contribution of left ventricular systolic dysfunction [13, 14].

Therefore, alternative approaches to characterize congestion beyond LVEF are needed. Biomarkers reflecting myocardial stress and systemic congestion, such as BNP and CA125, may provide a more biologically meaningful framework for risk stratification [4, 15, 16].

The primary aim of this study was to characterize patient subgroups defined by BNP and CA125 constellations, describe their clinical and echocardiographic correlations, and assess their association with all-cause mortality and heart failure rehospitalization. As a secondary objective, we explored their relationship with conventional LVEF categories.

## Methods

### Study design and patients

This is a retrospective observational study of a cohort of patients admitted to the Internal Medicine department of the Hospital Ramón y Cajal with the diagnosis of acute heart failure between July 2020 and May 2023.

Acute heart failure was defined as the rapid onset of symptoms and signs secondary to abnormal cardiac function, along with objective evidence of structural or functional cardiac abnormalities at rest, according to current guidelines [1]. An echocardiographic assessment of ejection fraction was performed either during hospitalization once the patient was clinically stable or, if available, using a study conducted within the previous year.

At our institution, patients presenting to the Emergency Department with acute heart failure are admitted through a structured clinical pathway that allocates patients to different departments according to their clinical profile. In general, older patients with a high burden of comorbidity and predominantly preserved ejection fraction are admitted to the Internal Medicine Department. Younger patients, those with lower comorbidity burden, and particularly those with reduced ejection fraction requiring advanced diagnostic evaluation or potential interventional management are preferentially admitted to the Cardiology Department. Patients presenting with multiple geriatric syndromes or complex geriatric needs are admitted to the Geriatrics Department. The present study includes exclusively patients admitted under the Internal Medicine Department.

This study was carried out in accordance with the Declaration of Helsinki and was approved by the ethics committee of the Hospital Ramón y Cajal (approval No. 052/18; 10 June 2020). All participants signed an informed consent form before participating in this study.

### Variables of study

Demographic data, medical history, vital signs, 12-lead electrocardiogram, laboratory data and treatments were determined during hospitalization. The following treatments were assessed: angiotensin converting enzyme inhibitors (ACEi), angiotensin receptor blockers (ARB), angiotensin receptor neprilysin inhibitors (ARNI), mineralocorticoid receptor antagonists (MRA), sodium-glucose co-transporter-2 inhibitors (SGLT2-i), beta blockers, and furosemide. Treatment decisions were made at the discretion of the treating physician in accordance with current guidelines [1].

### Echocardiographic parameters

Echocardiographic examinations were conducted after initial patient stabilization during hospitalization index by experienced operators from the local echocardiography laboratory. Cardiac chamber dimensions and systolic and diastolic function were measured in accordance with the current echocardiography guidelines [17].

Left ventricular ejection fraction was primarily assessed using the biplane Simpson method from apical two- and four-chamber views, following current guideline recommendations [17]. Visual estimation was reserved for cases with suboptimal image quality precluding reliable quantitative assessment.

In patients with atrial fibrillation, LVEF was measured in accordance with guideline recommendations [17] for irregular rhythms, averaging 3–5 consecutive cardiac cycles when feasible to minimize beat-to-beat variability. Quantitative assessment using the biplane Simpson method was preferred whenever image quality allowed.

A conventional EF cut-off of 50% was used to classify patients into two groups: those with heart failure with reduced ejection fraction (HFrEF, EF < 50%) and those with heart failure with preserved ejection fraction (HFpEF, EF ≥ 50%).

The analysis included the E/e′ ratio, left atrial volume index, left ventricular end-systolic and end-diastolic volumes, tricuspid annular plane systolic excursion (TAPSE), and systolic pulmonary artery pressure (sPAP).

### Clinical, biochemical and ultrasonographic parameters of congestion

At admission, clinical signs of congestion such as edema, pulmonary rales, and jugular vein distension were assessed.

Baseline blood tests were performed within 24 h of admission and analyzed in the local laboratory, including measurements of BNP and CA125, using the Roche BNP and CA125 assays, respectively.

Additionally, point-of-care ultrasound was used at admission to evaluate inferior vena cava diameter and collapsibility, as well as the presence of pulmonary B-lines and pleural effusion.

### Biomarker-defined patient subgroups

Four patient subgroups were defined according to cohort median values of BNP and CA125: Low BNP/Low CA125 (C1), High BNP/Low CA125 (C2), Low BNP/High CA125 (C3), and High BNP/High CA125 (C4).

BNP and CA125 were dichotomized using the median value of the overall cohort. Based on recent consensus documents on congestion assessment [4], these biomarker profiles were considered to reflect different pathophysiological patterns of congestion, ranging from relatively decongested states to combined myocardial and systemic congestion.

### Endpoints

The primary objective of the study was to characterize patient subgroups defined by BNP and CA125 constellations, including their clinical, echocardiographic, and prognostic correlates, specifically all-cause mortality and heart failure rehospitalization. Secondary objectives included: (1) the comparison of congestion-related features according to LVEF category (LVEF ≥ 50% vs. LVEF < 50%), and (2) the evaluation of the association between LVEF and all-cause mortality and heart failure rehospitalization.

### Statistical analysis

Continuous variables are presented as mean ± standard deviation (SD) or median with interquartile ranges (IQR), as appropriate, and categorical variables as percentages.

Baseline characteristics were primarily compared across biomarker-defined congestion phenotypes using the *χ*^2^ test for categorical variables and ANOVA or Kruskal–Wallis test for continuous variables, depending on data distribution. Comparisons according to LVEF category (≥ 50% vs. < 50%) were performed as secondary analyses using the same methods.

Time-to-event analyses for all-cause mortality and heart failure rehospitalization were performed using the Kaplan–Meier method, with differences between groups assessed by the log-rank test.

Cox proportional hazards regression models were used to evaluate the association between biomarker-defined congestion subgroups and all-cause mortality and heart failure rehospitalization. Cox model assumptions were verified using Schoenfeld residuals (proportional hazards assumption), Martingale and deviance residuals (functional form and goodness-of-fit), and dfbeta statistics (influential observations). No major violations were identified. Additional models were constructed to assess the association between LVEF category and the same outcomes. Results are reported as hazard ratios (HR) with 95% confidence intervals (CI).

Multivariable models were adjusted for clinically relevant covariates selected a priori based on prior evidence, including age, sex, systolic blood pressure, creatinine, hemoglobin, pleural effusion, peripheral edema, and LVEF. In models evaluating LVEF, BNP and CA125 were included as covariates. In contrast, in models evaluating biomarker-defined patient subgroups, BNP and CA125 were not included as continuous variables to avoid collinearity with the exposure definition. BNP and CA125 were also examined as continuous covariates in adjusted Cox models, and their interaction term was formally tested; these analyses are presented in Supplementary Table S2.

Additionally, LVEF was analyzed as a continuous variable using restricted cubic splines to explore potential non-linear associations with outcomes.

As a sensitivity analysis, a clinical congestion score was constructed based on the presence of three signs: jugular venous distension, pulmonary crackles, and peripheral edema (range 0–3). This score was evaluated in patients with complete data on all three variables, and its association with all-cause mortality and heart failure rehospitalization was assessed using adjusted Cox models.

We set a two-sided *p*-value of < 0.05 as the threshold for statistical significance. Stata 18 (StataCorp. 2023. Stata Statistical Software: Release 18. College Station, TX: StataCorp LLC.) was used for these analyses.

## Results

### Baseline characteristics

A total of 830 patients were included in the study. The median age was 87 years (IQR: 83–90), and women represented 65.6% of the cohort. A total of 677 patients (81.7%) had preserved ejection fraction (LVEF ≥ 50%) and 153 (18.3%) had reduced ejection fraction (LVEF < 50%).

### Baseline characteristics by biomarker-defined patient subgroups

C1 (Low BNP/Low CA125) represented the least congested group, with the lowest rates of atrial fibrillation (52.9%), peripheral edema (65.4%), and pleural effusion (31.9%), the highest prevalence of preserved ejection fraction (89.5%), and the best renal function (eGFR 47.3 mL/min/1.73m^2^). C2 (High BNP/Low CA125) was characterized by lower LVEF (58%), higher rates of reduced ejection fraction (24.7%), and reduced TAPSE. C3 (Low BNP/High CA125) showed the highest rates of peripheral edema (70.4%) and pleural effusion (48.7%), with relatively preserved ejection fraction (88.9%) and renal function (eGFR 48.8 mL/min/1.73m^2^). C4 (High BNP/High CA125) had the most adverse profile, with the oldest age (median 88 years), highest rates of atrial fibrillation (67.7%) and pleural effusion (48.7%), worst renal function (eGFR 41.7 mL/min/1.73m^2^), and highest mortality (59.3%).

Other comorbidities, including hypertension, diabetes, dyslipidemia, COPD, and ischemic etiology, were broadly similar across groups. NYHA functional class distribution was comparable across subgroups. Laboratory data showed that renal dysfunction was most pronounced in C4, with higher creatinine and lower eGFR compared with other groups (*p* < 0.001). Electrolytes and hemoglobin levels were similar across subgroups.

Regarding treatments, ACEi/ARB and SGLT2 inhibitor use differed modestly between groups (*p* = 0.043 and *p* = 0.013, respectively). Loop diuretic equivalent dose was slightly higher in C3 and C4 (*p* = 0.004).

Baseline characteristics are summarized in Table 1.

**Table 1:** Baseline characteristics according to biomarker-defined congestion phenotype

Variables	Total (*n* = 830)	C1: Low BNP/Low CA125 (*n* = 257)	C2: High BNP/Low CA125 (*n* = 158)	C3: Low BNP/High CA125 (*n* = 189)	C4: High BNP/High CA125 (*n* = 226)	*p* value
Demographics and medical history						
Age, years	87 (83–90)	86 (81–89)	88 (85–91)	87 (83–90)	88 (83–91)	< 0.001
Female sex, *n* (%)	544 (65.6)	173 (67.2)	102 (64.6)	122 (64.5)	147 (65.3)	0.917
Hypertension, *n* (%)	741 (89.3)	235 (91.4)	140 (88.6)	164 (86.7)	202 (89.4)	0.463
Diabetes mellitus, *n* (%)	325 (39.2)	110 (42.8)	47 (29.7)	78 (41.3)	90 (39.8)	0.053
Dyslipidemia, *n* (%)	497 (59.9)	158 (61.5)	87 (55.1)	114 (60.3)	138 (61.1)	0.584
COPD, *n* (%)	181 (21.8)	58 (22.6)	30 (19.0)	40 (21.2)	53 (23.5)	0.746
Atrial fibrillation, *n* (%)	493 (59.4)	136 (52.9)	82 (51.9)	122 (64.6)	153 (67.7)	0.001
Ischemic etiology, *n* (%)	197 (23.7)	55 (21.4)	33 (20.9)	53 (28.0)	56 (24.8)	0.314
Valvular heart disease, *n* (%)	233 (28.1)	62 (24.1)	41 (26.0)	58 (30.1)	72 (31.9)	0.205
First HF hospitalization, *n* (%)*	[CORREGIR]	55 (39.3)	25 (26.9)	28 (30.1)	35 (25.9)	0.076
NYHA functional class						
Class I	31 (3.7)	14 (5.5)	5 (3.2)	5 (2.7)	7 (3.1)	
Class II	559 (67.4)	172 (67.0)	114 (72.2)	124 (65.6)	149 (66.0)	
Class III	230 (27.7)	69 (26.9)	37 (23.4)	57 (30.2)	67 (29.7)	
Class IV	10 (1.2)	2 (0.8)	2 (1.3)	3 (1.6)	3 (1.3)	0.721
Vital signs						
SBP, mmHg	134 (118–150)	134 (120–148)	135 (118–152)	134 (120–149)	131 (117–150)	0.779
DBP, mmHg	72 (62–85)	72 (63–80)	71 (60–84)	71 (61–83)	75 (65–89)	0.380
Heart rate, bpm	80 (68–94)	79 (68–93)	78 (68–95)	82 (70–92)	84 (72–96)	0.620
Clinical signs of congestion						
Peripheral edema, *n* (%)	555 (66.9)	168 (65.4)	90 (57.0)	133 (70.4)	164 (72.6)	< 0.001
Pulmonary rales, *n* (%)^†^	373 (76.3)	114 (72.2)	78 (74.3)	69 (75.8)	112 (83.0)	0.167
Jugular venous distension, *n* (%)^†^	154 (31.5)	58 (36.7)	39 (37.2)	18 (19.8)	39 (28.9)	0.021
Echocardiography						
LVEF, %	61 (52.6–67.8)	63 (57.0–69.7)	58 (47.4–65.3)	63 (56.0–68.6)	59 (56.0–65.6)	< 0.001
LVEF ≥ 50%, *n* (%)	677 (81.6)	230 (89.5)	119 (75.3)	168 (88.9)	160 (70.8)	< 0.001
E/e' ratio	14.9 (11.2–19.5)	13.7 (10.4–17.5)	15.0 (11.4–20.3)	15.0 (11.4–18.9)	16.0 (12.2–21.5)	0.007
Left atrial volume index, mL/m^2^	48.5 (37.6–61.1)	45.0 (34.2–55.5)	46.4 (36.3–65.2)	49.5 (37.2–63.1)	50.6 (41.9–63.8)	0.003
LVEDD, mm	43 (35–49)	43 (36–49)	42 (34–50)	43 (36–48)	44 (35–49)	0.960
LVESD, mm	25 (20–31)	24 (20–30)	25 (19–33)	[CORREGIR]	26 (21–33)	0.217
TAPSE, mm	20 (17–23)	20 (18–24)	19 (17–23)	20 (18–23)	19 (16–23)	< 0.001
sPAP, mmHg	47 (37.9–59.5)	45.9 (35.5–56.2)	47.5 (41.0–55.0)	45.0 (37.6–58.9)	50.0 (40.0–60.0)	0.190
Point-of-care ultrasound						
Dilated IVC, *n* (%)^†^	148 (41.5)	49 (46.7)	30 (41.1)	31 (43.7)	38 (35.2)	0.381
Pleural effusion, *n* (%)	328 (39.5)	82 (31.9)	44 (27.9)	92 (48.7)	110 (48.7)	< 0.001
Laboratory data						
Creatinine, mg/dL	1.2 (0.9–1.7)	1.1 (0.8–1.5)	1.3 (0.9–1.8)	1.1 (0.8–1.6)	1.4 (1.1–1.8)	< 0.001
eGFR, mL/min/1.73m^2^	44.5 (32.3–62.6)	47.3 (35.2–67.7)	43.1 (29.0–56.8)	48.8 (36.0–69.0)	41.7 (30.7–55.8)	< 0.001
Sodium, mEq/L	139 (136–142)	139 (136–141)	139 (136–142)	139 (135–142)	139 (136–142)	0.740
Potassium, mEq/L	4.5 (4.2–4.9)	4.5 (4.2–4.9)	4.6 (4.1–4.9)	4.5 (4.1–4.9)	4.5 (4.2–5.0)	0.065
Chloride, mEq/L	102 (98–107)	102 (98–105)	103 (98–107)	102 (98–106)	103 (99–107)	0.081
Hemoglobin, g/dL	12.0 (10.6–13.4)	12.2 (10.6–13.6)	12.0 (10.6–13.6)	12.0 (10.7–13.1)	11.8 (10.5–13.3)	0.797
BNP, pg/mL	585 (326–985)	315 (194–497)	967 (763–1334)	378 (261–518)	1077 (815–1649)	< 0.001
CA125, U/mL	56.8 (26.6–119.8)	22.6 (14.3–38.0)	33.0 (22.3–44.4)	107.8 (77.2–170.7)	131.0 (93.0–200.2)	< 0.001
Treatment at admission						
ACEi/ARB, *n* (%)	431 (51.9)	150 (58.4)	77 (48.7)	100 (52.9)	104 (46.0)	0.043
Sacubitril/valsartan, *n* (%)	28 (3.4)	4 (1.6)	10 (6.3)	4 (2.1)	10 (4.4)	0.039
Beta-blocker, *n* (%)	462 (55.7)	130 (50.6)	89 (56.3)	105 (55.6)	138 (61.1)	0.146
MRA, *n* (%)	89 (10.7)	29 (11.3)	13 (8.2)	28 (14.8)	19 (8.4)	0.128
SGLT2i, *n* (%)	309 (37.2)	90 (35.0)	68 (43.0)	55 (29.1)	96 (42.5)	0.013
Furosemide, *n* (%)	557 (67.1)	165 (64.2)	99 (62.7)	139 (73.5)	154 (68.1)	0.109
Furosemide equivalent dose, mg/day	40 (40–80)	40 (40–80)	40 (40–60)	40 (40–80)	40 (40–80)	0.004
Thiazides, *n* (%)	113 (13.6)	39 (15.2)	23 (14.6)	28 (14.8)	23 (10.2)	0.369
Clinical outcomes						
HF rehospitalization, *n* (%)	301 (36.3)	87 (33.9)	54 (34.2)	78 (41.3)	82 (36.3)	0.393
All-cause mortality, *n* (%)	418 (50.4)	104 (40.5)	78 (49.4)	102 (54.0)	134 (59.3)	< 0.001

Data are presented as median (IQR) or *n* (%)

*ACEi* angiotensin-converting enzyme inhibitor, *ARB* angiotensin receptor blocker, *BNP* B-type natriuretic peptide, *CA125* carbohydrate antigen 125, *COPD* chronic obstructive pulmonary disease, *DBP* diastolic blood pressure, *eGFR* estimated glomerular filtration rate, *HF* heart failure, *IVC* inferior vena cava, *LVEDD* left ventricular end-diastolic diameter, *LVEF* left ventricular ejection fraction, *LVESD* left ventricular end-systolic diameter, *MRA* mineralocorticoid receptor antagonist, *NYHA* New York Heart Association, *SBP* systolic blood pressure, *SGLT2i* sodium-glucose cotransporter 2 inhibitor, *sPAP* systolic pulmonary artery pressure, *TAPSE* tricuspid annular plane systolic excursion

*Calculated in patients with available data

^†^Calculated in patients with available data

### Association between biomarker-defined patient subgroups and clinical outcomes

#### All-cause mortality

During a median follow-up of 310 days (IQR 62–543), 418 patients (50.4%) died.

All-cause mortality increased progressively across biomarker-defined phenotypes (C1 40.5%, C2 49.4%, C3 54.0%, C4 59.3%; *p* < 0.001).

**Fig. 1:**
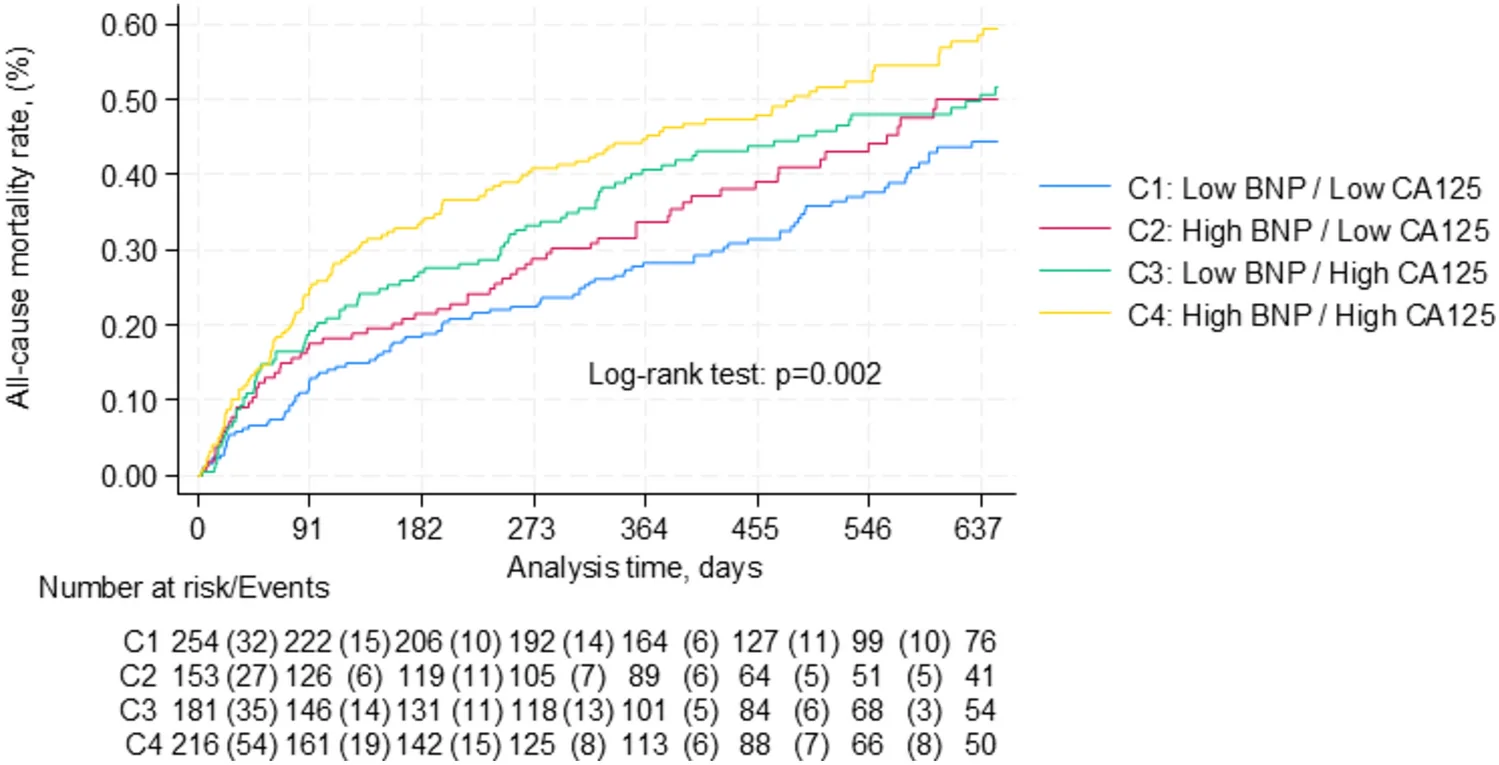
Kaplan–Meier curves for all-cause mortality according to biomarker-defined congestion phenotype. Kaplan–Meier survival curves showing cumulative all-cause mortality across the four biomarker-defined congestion phenotypes: C1 (Low BNP/Low CA125), C2 (High BNP/Low CA125), C3 (Low BNP/High CA125), and C4 (High BNP/High CA125). Differences between groups were assessed using the log-rank test. *BNP* B-type natriuretic peptide, *CA125* carbohydrate antigen 125

Kaplan–Meier curves demonstrated significant differences across phenotypes, with higher mortality in C3 and C4 (log-rank *p* = 0.002) (Fig. 1).

In multivariable Cox analysis, only patients in C4 (High BNP/High Ca125) showed a significantly higher risk of all-cause mortality compared with C1 (HR 1.48, 95% CI 1.11–1.99; *p* = 0.008). C2 and C3 were not independently associated with mortality. (Table 3).

#### Heart failure hospitalizations

During the same follow-up, 301 (36.3%) patients presented rehospitalization. Heart failure rehospitalization rates were similar across subgroups (C1 33.9%, C2 34.2%, C3 41.3%, C4 36.3%), with a non-significant trend toward higher rates in C3 and C4.

**Fig. 2:**
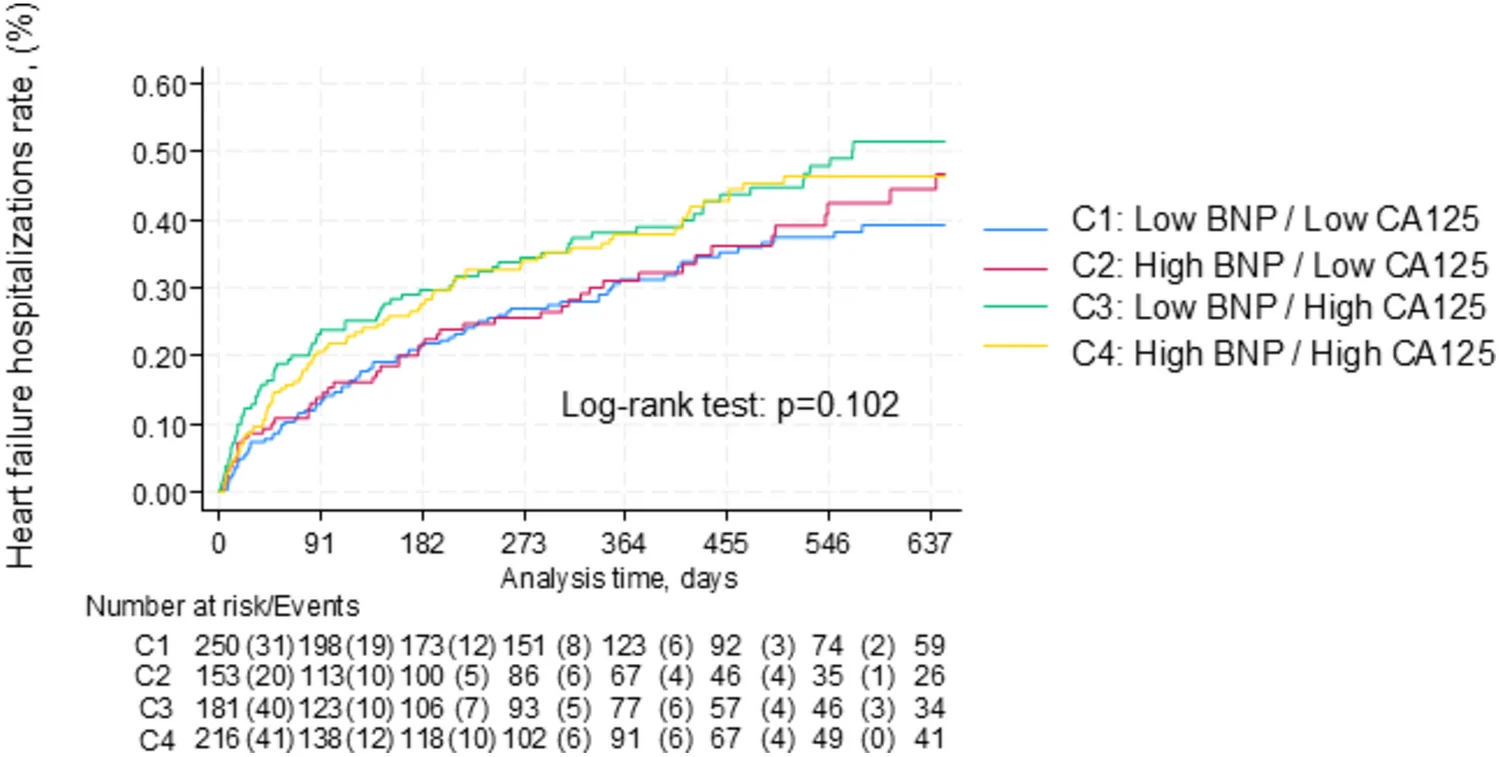
Kaplan–Meier curves for heart failure rehospitalization according to biomarker-defined congestion phenotype. Kaplan–Meier curves showing cumulative heart failure rehospitalization-free survival across the four biomarker-defined congestion phenotypes: C1 (Low BNP/Low CA125), C2 (High BNP/Low CA125), C3 (Low BNP/High CA125), and C4 (High BNP/High CA125). Differences between groups were assessed using the log-rank test. *BNP* B-type natriuretic peptide, *CA125* carbohydrate antigen 125

Kaplan–Meier analysis showed visually divergent curves across biomarker-defined subgroups, with higher event rates in C3 and C4, although differences did not reach statistical significance (log-rank *p* = 0.109) (Fig. 2).

In adjusted Cox models, compared with C1, only C3 (Low BNP/High CA125) was significantly associated with a higher risk of heart failure rehospitalization (HR 1.45, 95% CI 1.03–2.03; *p* = 0.031). C2 and C4 did not reach statistical significance for this outcome. (Supplementary Table S1).

### LVEF-based analysis

#### Congestion profile according to LVEF

Clinical signs of congestion were similar across LVEF categories, with no meaningful differences in peripheral edema, pulmonary rales, or jugular venous distension. NYHA class distribution was also comparable, while systolic blood pressure was slightly lower in patients with LVEF < 50%.

Echocardiographic assessment showed expected structural differences, with larger ventricular dimensions and lower TAPSE in patients with LVEF < 50%. In contrast, point-of-care ultrasound findings were similar across groups.

Biomarker levels differed between categories, with higher BNP and CA125 concentrations in patients with LVEF < 50%.

Baseline characteristics across LVEF Category are summarized in Table 2.

**Table 2:** Baseline characteristics according to LVEF category

Variables	HFpEF LVEF ≥ 50% (*n* = 677)	HFrEF LVEF < 50% (*n* = 153)	*p* value
Demographics and medical history			
Age, years	87 (83–90)	87 (81–90)	0.275
Female sex, *n* (%)	477 (70.5)	67 (44.1)	< 0.001
Hypertension, *n* (%)	605 (89.4)	136 (88.9)	0.864
Diabetes mellitus, *n* (%)	195 (28.8)	40 (26.1)	0.510
Dyslipidemia, *n* (%)	403 (59.5)	94 (61.4)	0.663
COPD, *n* (%)	140 (20.7)	41 (26.8)	0.098
Atrial fibrillation, *n* (%)	407 (60.1)	86 (56.2)	0.374
Ischemic etiology, *n* (%)	138 (20.4)	59 (38.6)	< 0.001
Valvular heart disease, *n* (%)	192 (28.4)	41 (26.8)	0.698
First HF hospitalization, *n* (%)	394 (58.4)	73 (48.0)	0.020
NYHA functional class			
Class I	23 (3.4)	8 (5.2)	
Class II	461 (68.1)	98 (64.0)	
Class III	184 (27.2)	46 (30.1)	
Class IV	9 (1.3)	1 (0.6)	0.518
Vital signs			
SBP, mmHg	134 (120–150)	132 (111–146)	0.035
DBP, mmHg	72 (62–84)	73 (64–89)	0.324
Heart rate, bpm	80 (70–93)	80 (69–100)	0.378
Clinical signs of congestion			
Peripheral edema, *n* (%)	470 (69.4)	94 (61.4)	0.056
Pulmonary rales, *n* (%)^†^	305 (81.5)	68 (78.2)	0.469
Jugular venous distension, *n* (%)^†^	103 (27.5)	23 (26.4)	0.835
Echocardiography			
LVEF, %	64 (58.6–69.7)	40 (34.8–45.1)	< 0.001
E/e' ratio	14.7 (10.9–19.5)	15.7 (12.1–19.3)	0.452
Left atrial volume index, mL/m^2^	46.7 (37.0–60.0)	52.0 (39.0–66.2)	0.039
LVEDD, cm	4.2 (3.5–4.8)	4.8 (3.8–5.4)	< 0.001
LVESD, cm	2.4 (1.9–2.9)	3.3 (2.5–3.9)	< 0.001
TAPSE, cm	2.0 (1.8–2.4)	1.8 (1.5–2.1)	< 0.001
sPAP, mmHg	47.5 (38.4–59.9)	45.0 (36.0–55.0)	0.406
Point-of-care ultrasound			
Dilated IVC, *n* (%)^†^	147 (50.8)	38 (55.9)	0.456
Pulmonary B-lines, *n* (%)^†^	120 (42.2)	27 (41.5)	0.916
Pleural effusion, *n* (%)	269 (39.7)	59 (38.6)	0.789
Laboratory data			
Creatinine, mg/dL	1.2 (0.9–1.6)	1.3 (1.0–1.7)	0.022
eGFR, mL/min/1.73m^2^	45.0 (32.6–63.4)	41.8 (31.1–58.0)	0.079
Sodium, mEq/L	139 (136–142)	139 (135–141)	0.291
Potassium, mEq/L	4.5 (4.1–4.9)	4.5 (4.2–5.0)	0.444
Chloride, mEq/L	100 (95–103)	100 (96–103)	0.171
Hemoglobin, g/dL	11.9 (10.5–13.3)	12.5 (11.0–13.8)	0.003
BNP, pg/mL	549 (306–860)	945 (585–1434)	< 0.001
CA125, U/mL	55.4 (25.0–114.2)	69.6 (33.4–132.9)	0.010
Total proteins, g/dL	6.2 (5.9–6.6)	6.1 (5.7–6.4)	0.028
Albumin, g/dL	3.1 (2.8–3.3)	3.1 (2.7–3.3)	0.739
Treatment at admission			
ACEi/ARB, *n* (%)	360 (53.2)	71 (46.4)	0.130
Sacubitril/valsartan, *n* (%)	2 (0.3)	26 (17.0)	< 0.001
Beta-blocker, *n* (%)	375 (55.4)	87 (56.9)	0.741
MRA, *n* (%)	68 (10.0)	21 (13.7)	0.184
SGLT2i, *n* (%)	240 (35.4)	69 (45.1)	0.026
Quadruple therapy (GDMT4), *n* (%)	8 (1.2)	17 (11.1)	< 0.001
Furosemide, *n* (%)	456 (67.4)	101 (66.0)	0.749
Furosemide equivalent dose, mg/day	40 (40–80)	60 (40–80)	0.249
Thiazides, *n* (%)	96 (14.2)	17 (11.1)	0.317
Clinical outcomes			
HF rehospitalization, *n* (%)	251 (37.1)	50 (32.7)	0.304
All-cause mortality, *n* (%)	334 (49.3)	84 (54.9)	0.213

Data are presented as median (IQR) or *n* (%)

*ACEi* angiotensin-converting enzyme inhibitor, *ARB* angiotensin receptor blocker, *BNP* B-type natriuretic peptide, *CA125* carbohydrate antigen 125, *COPD* chronic obstructive pulmonary disease, *DBP* diastolic blood pressure, *eGFR* estimated glomerular filtration rate, *GDMT4* quadruple guideline-directed medical therapy (ARNi + beta-blocker + MRA + SGLT2i), *HF* heart failure, *HFpEF* heart failure with preserved ejection fraction, *HFrEF*, heart failure with reduced ejection fraction, *IVC* inferior vena cava, *LVEDD* left ventricular end-diastolic diameter, *LVEF* left ventricular ejection fraction, *LVESD* left ventricular end-systolic diameter, *MRA* mineralocorticoid receptor antagonist, *NYHA* New York Heart Association, *SBP* systolic blood pressure, *SGLT2i* sodium-glucose cotransporter 2 inhibitor, *sPAP* systolic pulmonary artery pressure, *TAPSE* tricuspid annular plane systolic excursion

^†^Calculated in patients with available data

#### Association between LVEF and outcomes

**Fig. 3:**
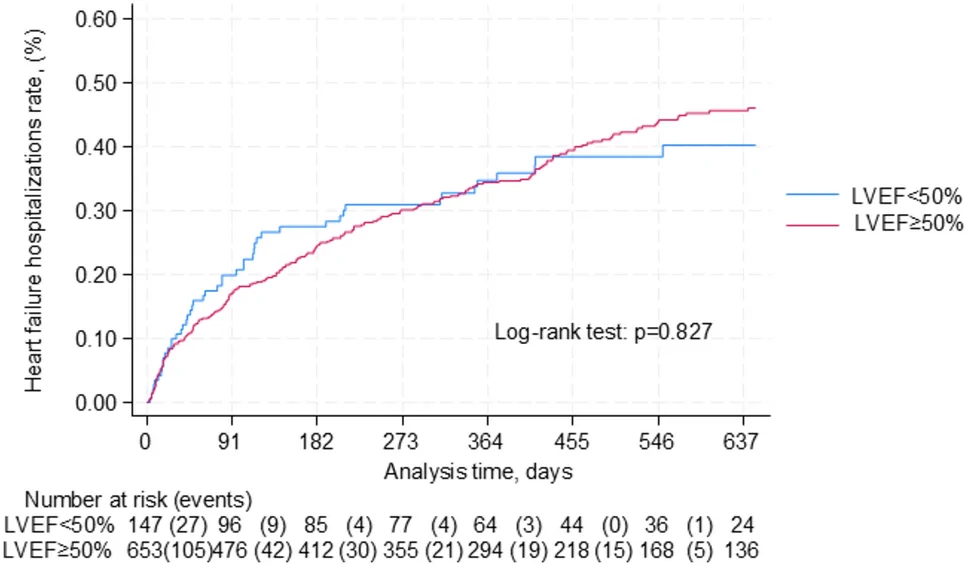
Kaplan–Meier curves for heart failure rehospitalization according to LVEF category. Kaplan–Meier curves showing cumulative heart failure rehospitalization-free survival in patients with heart failure with preserved ejection fraction (HFpEF, LVEF ≥ 50%) and heart failure with reduced ejection fraction (HFrEF, LVEF < 50%). Differences between groups were assessed using the log-rank test. *LVEF* left ventricular ejection fraction

Rehospitalization rates were similar across LVEF categories, with no significant differences in Kaplan–Meier analysis (Fig. 3).

In multivariable Cox models, higher LVEF was modestly associated with increased rehospitalization risk, although this finding should be interpreted cautiously (Table 3).

**Table 3:** Multivariable Cox regression analysis for all-cause mortality

Variable	Hazard ratio (HR)	95% CI	*p* value
Clinical and demographic covariates			
Age (per year)	1.05	1.03–1.08	< 0.001
Female sex	0.73	0.58–0.93	0.010
Systolic BP (per mmHg)	1.00	1.00–1.00	0.524
Creatinine (per mg/dL)	1.00	0.99–1.01	0.632
Hemoglobin (per g/dL)	0.97	0.93–1.02	0.299
Pleural effusion	1.13	0.90–1.42	0.284
Peripheral edema	0.88	0.70–1.11	0.275
LVEF (per %)	1.00	0.99–1.01	0.621
Biomarker-defined congestion phenotype (ref: C1)			
C2—High BNP/Low CA125	1.02	0.72–1.43	0.924
C3—Low BNP/High CA125	1.26	0.92–1.72	0.151
C4—High BNP/High CA125	1.48	1.11–1.99	0.008

Adjusted for age, sex, systolic blood pressure, creatinine, hemoglobin, pleural effusion, peripheral edema, and LVEF. Biomarker phenotypes: C1 (Low BNP/Low CA125, reference); C2 (High BNP/Low CA125); C3 (Low BNP/High CA125); C4 (High BNP/High CA125)

*BP* blood pressure, *CI* confidence interval, *HR* hazard ratio, *LVEF* left ventricular ejection fraction

**Fig. 4:**
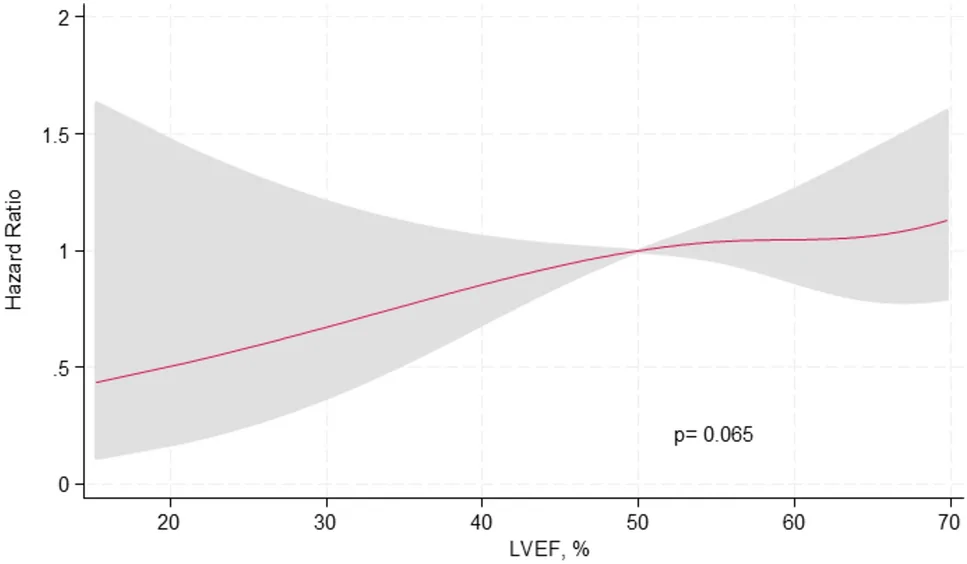
Association between LVEF as a continuous variable and the risk of heart failure rehospitalization. Restricted cubic spline analysis showing the association between left ventricular ejection fraction (LVEF) modeled as a continuous variable and the hazard ratio for heart failure rehospitalization, adjusted for age, sex, systolic blood pressure, creatinine, hemoglobin, pleural effusion, and peripheral edema. The solid line represents the estimated hazard ratio and the shaded area the 95% confidence interval. The reference value is set at the median LVEF of the cohort. *LVEF* left ventricular ejection fraction

When LVEF was modelled using restricted cubic splines, a modest, non-linear trend was observed, with lower estimated risk at reduced LVEF values and a slight increase at higher LVEF levels; however, the overall spline term did not reach conventional statistical significance (*p* = 0.065), indicating that strong evidence for non-linearity was lacking (Fig. 4).

**Fig. 5:**
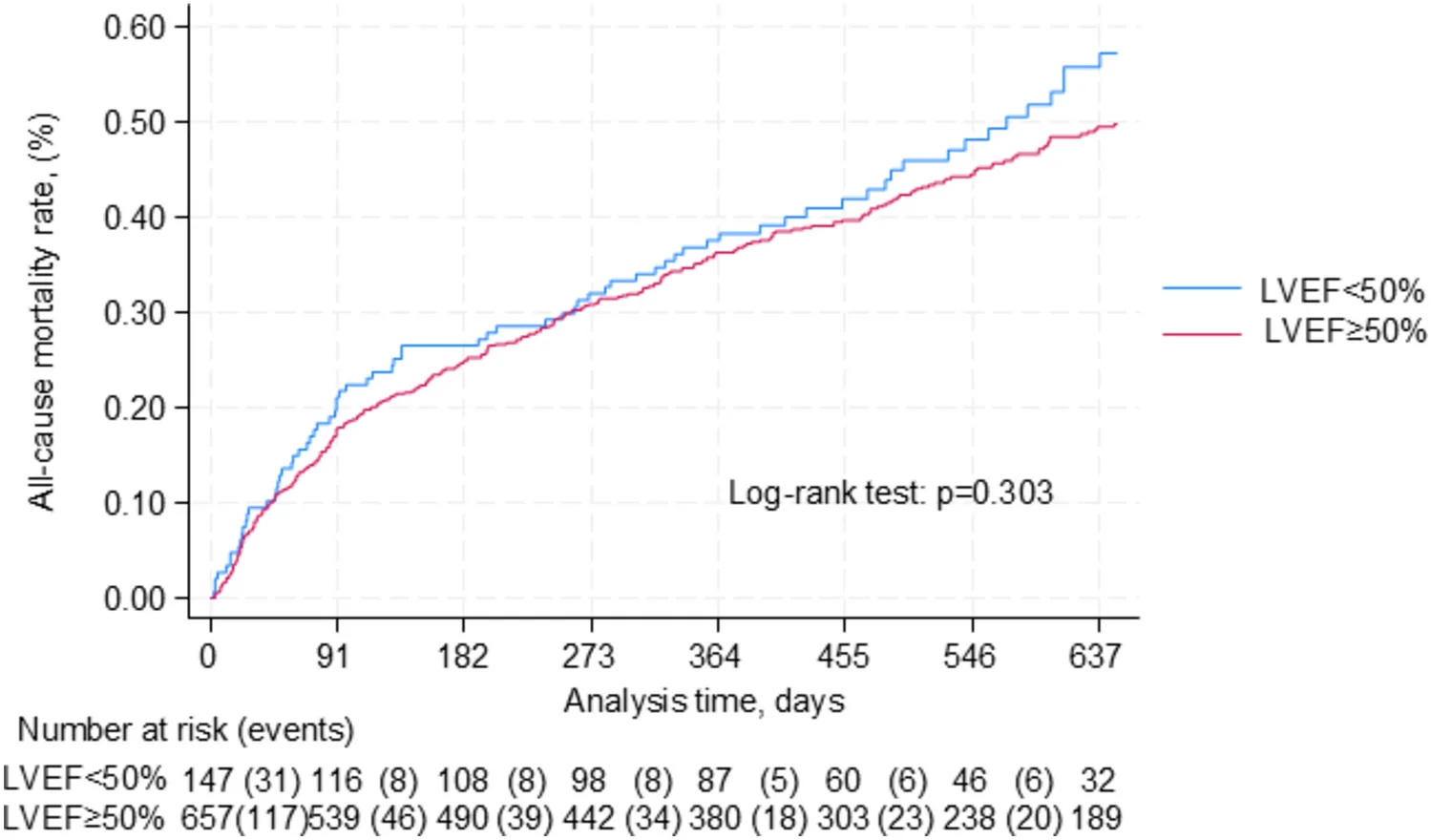
Kaplan–Meier curves for all-cause mortality according to LVEF category. Kaplan–Meier survival curves showing cumulative all-cause mortality in patients with heart failure with preserved ejection fraction (HFpEF, LVEF ≥ 50%) and heart failure with reduced ejection fraction (HFrEF, LVEF < 50%). Differences between groups were assessed using the log-rank test. *LVEF* left ventricular ejection fraction

All-cause mortality did not differ between LVEF groups, and LVEF was not independently associated with mortality in adjusted models (Fig. 5).

**Fig. 6:**
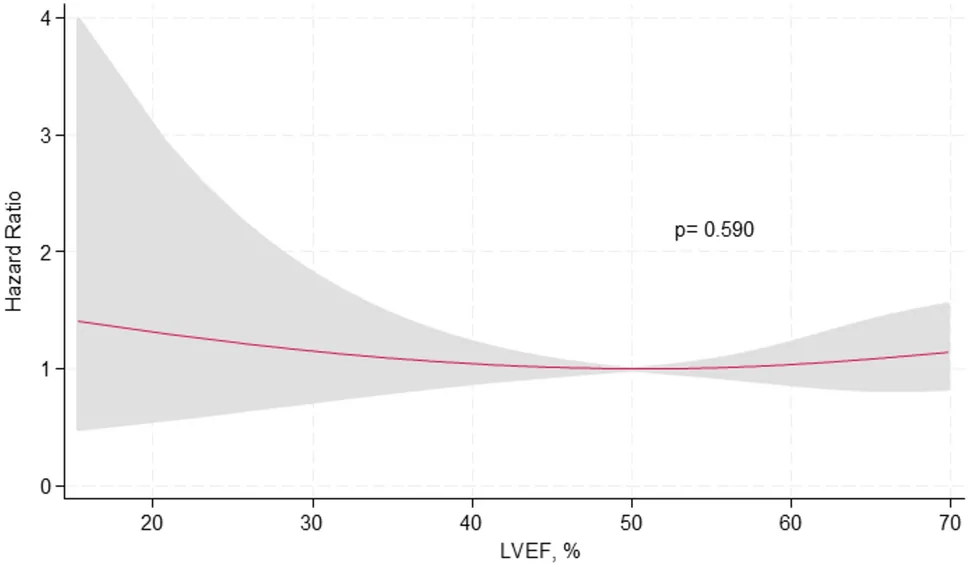
Association between LVEF as a continuous variable and the risk of all-cause mortality. Restricted cubic spline analysis showing the association between left ventricular ejection fraction (LVEF) modeled as a continuous variable and the hazard ratio for all-cause mortality, adjusted for age, sex, systolic blood pressure, creatinine, hemoglobin, pleural effusion, and peripheral edema. The solid line represents the estimated hazard ratio and the shaded area the 95% confidence interval. The reference value is set at the median LVEF of the cohort. *LVEF* left ventricular ejection fraction

When LVEF was modelled using restricted cubic splines, no meaningful association with the outcome was observed, and the overall spline term was not statistically significant (*p *= 0.590), indicating no evidence of a non-linear relationship (Fig. 6).

### Sensitivity analysis

In a sensitivity analysis restricted to patients with complete clinical data (*n *= 489), a composite clinical congestion score integrating jugular venous distension, pulmonary crackles and edema, was not independently associated with mortality (HR 0.962, *p* = 0.703) or rehospitalization (HR 1.001, *p* = 0.996) in adjusted models including BNP and CA125.

## Discussion

In this large cohort of very old patients hospitalized with acute heart failure, we characterized patient subgroups defined by BNP and CA125 constellations that provided prognostic stratification beyond conventional LVEF classification. While clinical signs of congestion and outcomes were largely similar across LVEF categories, combined elevation of BNP and CA125 was independently associated with increased mortality and isolated CA125 elevation was associated with higher rehospitalization risk.

These findings highlight the limited prognostic discrimination of LVEF in this population and suggest that integrating BNP and CA125 may complement conventional LVEF classification for risk stratification in very old patients with acute heart failure.

### Study population and interpretation of LVEF findings

An important aspect to consider when interpreting our findings is the selection of the study population. At our institution, patients with acute heart failure are allocated to different departments according to their clinical profile. As a result, the present cohort includes exclusively patients admitted under the Internal Medicine Department, who are typically older, more multimorbid, and more frequently present with preserved ejection fraction. In contrast, younger patients and those with more severe systolic dysfunction are more often managed in Cardiology units.

Therefore, this cohort does not represent the full spectrum of acute heart failure, but rather reflects a real-world geriatric population with high comorbidity burden. This selection may partly explain the predominance of HFpEF and the limited prognostic discrimination of LVEF observed in our study, and our findings should be interpreted within this specific clinical context.

The absence of a clear association between LVEF and outcomes in our study should be interpreted with caution. Due to the structured allocation of patients at our institution, individuals with reduced ejection fraction, particularly those with more advanced systolic dysfunction, may have been preferentially managed in Cardiology units, and thus underrepresented in our cohort. In addition, in very elderly populations, prognosis is strongly influenced by frailty, multimorbidity, and systemic factors, which may attenuate the relative contribution of LVEF.

Therefore, our findings should not be interpreted as indicating a lack of prognostic relevance of LVEF in acute heart failure overall, but rather as reflecting its limited discriminative value within this specific geriatric population.

### Biomarker-defined patient subgroups and pathophysiology

Beyond ejection fraction classification, patient subgroups defined by BNP and CA125 constellations provided a more nuanced characterization of disease severity and risk. Notably, different biomarker patterns were associated with distinct clinical trajectories. Isolated elevation of CA125 was associated with a higher risk of rehospitalization, whereas combined elevation of BNP and CA125 was independently associated with increased mortality.

These findings likely reflect different pathophysiological mechanisms of congestion. CA125 has been linked to systemic congestion, serosal inflammation, and fluid accumulation [16, 18], which may predispose to recurrent decompensation and rehospitalization. In contrast, BNP reflects myocardial wall stress and intracardiac pressure [19]. The combination of both markers may therefore capture a more advanced stage of disease characterized by both hemodynamic stress and systemic congestion, explaining its strong association with mortality [15].

Taken together, these results support the concept that congestion is a multidimensional process, and that biomarker-based profiling may better capture its biological complexity than clinical signs alone [15]. The absence of a significant association between C4 and rehospitalization may reflect a competing risk of death in this high-risk group. Patients with combined biomarker elevation experience higher early mortality, reducing their time at risk for rehospitalization. In contrast, C3, characterized by isolated systemic congestion with relatively preserved myocardial stress, may represent a phenotype at higher risk of recurrent decompensation, consistent with its significant association with rehospitalization. The limited prognostic value of clinical signs for rehospitalization is consistent with prior evidence suggesting that biomarkers better capture the biological trajectory underlying recurrent congestion, particularly in multimorbid elderly patients.

### LVEF as a secondary and limited classifier

Although LVEF remains a cornerstone of heart failure classification [1], our findings reinforce its limited prognostic value in very old patients with acute heart failure. Clinical signs of congestion and outcomes were largely similar across LVEF categories, consistent with previous studies showing comparable hemodynamic congestion across the EF spectrum [11, 20].

Importantly, LVEF was not independently associated with mortality and showed only a modest association with rehospitalization. Similar observations have been reported in prior cohorts, where outcomes in acute heart failure were not significantly different across ejection fraction groups [21, 22]. Recent data further suggest that the prognostic contribution of LVEF may be attenuated when etiological and comorbidity factors are considered across the ejection fraction spectrum [23].

In this context, reliance on LVEF alone may be insufficient to capture the complexity of acute heart failure in very old patients.

### Geriatric context: frailty, multimorbidity, biological vulnerability

The advanced age of this cohort underscores that these findings apply to a real-world population of very old and multimorbid patients. In this setting, prognosis is often driven by biological vulnerability, systemic congestion, and comorbidity burden rather than by left ventricular systolic function alone.

Frailty, although not formally assessed, is highly prevalent in this population and likely contributes substantially to outcomes [14]. Beyond chronological age, frailty reflects reduced physiological reserve and impaired response to stressors, which may influence both congestion tolerance and recovery after decompensation [14].

These factors may attenuate the prognostic relevance of LVEF and further support the need for multidimensional risk stratification approaches.

The very advanced age of our cohort (median 87 years) highlights the relevance of these findings in a population that is frequently underrepresented in randomized clinical trials, yet represents a substantial proportion of patients hospitalized with acute heart failure in real-world practice.

### Clinical implications

Our findings have several clinical implications. First, they suggest that biomarker-based congestion profiling may provide incremental prognostic information beyond traditional LVEF classification. In very old patients, where clinical presentation is often heterogeneous and less discriminatory, objective biomarkers such as BNP and CA125 may better reflect the underlying biological burden of disease.

Second, biomarker-defined patient subgroups may help identify patients at differential risk of rehospitalization and mortality, potentially informing follow-up intensity and post-discharge management. Patients with isolated CA125 elevation may benefit from closer monitoring for recurrent congestion, whereas those with combined biomarker elevation may require more intensive global management strategies.

Third, the patient subgroups were derived using median splits of BNP and CA125, representing a pragmatic and clinically interpretable approach. However, this method does not rely on validated biological thresholds and may oversimplify the continuous relationship between biomarker levels and risk. Therefore, these phenotypes should be considered exploratory and hypothesis-generating, and further studies are needed to validate this classification.

Finally, these findings support a shift toward a multiparametric approach integrating clinical, imaging, and biomarker data, particularly in geriatric populations where traditional classifications may be insufficient.

### Pathophysiological considerations

Although HFpEF and HFrEF differ in chronic pathophysiology [5, 24], acute decompensation appears to converge toward a common final pathway of elevated filling pressures [3]. This may explain the similar clinical presentation across LVEF categories observed in our study.

However, the distinct biomarker and echocardiographic patterns suggest that the mechanisms underlying congestion may still differ between phenotypes, with a greater contribution of fluid redistribution and vascular stiffness in HFpEF, and more pronounced structural remodeling in HFrEF [5, 24]. These interpretations are hypothesis-generating and warrant further investigation.

### Limitations

This study has several limitations. First, its retrospective, single-center design may limit generalizability. Second, the study population reflects a selected cohort of very old and multimorbid patients admitted to the Internal Medicine Department, which may underrepresent patients with more severe systolic dysfunction managed in cardiology units.

Third, congestion assessment was based on parameters measured at admission, without serial evaluation of treatment response or residual congestion. Fourth, invasive hemodynamic measurements were not available.

Fifth, biomarker-defined patient subgroups were based on median values of BNP and CA125 and should therefore be considered exploratory and hypothesis-generating. Finally, frailty was not formally assessed, which is an important limitation in this geriatric population.

## Conclusions

In this cohort of very old adults with acute heart failure, biomarker-defined subgroups based on BNP and CA125 provided prognostic stratification beyond LVEF categories. Combined elevation of BNP and CA125 was independently associated with increased mortality, while LVEF was not. These findings are hypothesis-generating and suggest that integrating BNP and CA125 may complement conventional LVEF classification for risk stratification in geriatric and multimorbid populations.

## Supplementary Information

Below is the link to the electronic supplementary material.

Supplementary file1 (DOCX 26 KB)

Supplementary file2 (DOCX 31 KB)

## Data Availability

The data that support the findings of this study are not publicly available due to privacy and ethical restrictions. Datamay be available from the corresponding author (Pau Llàcer, paullacer@hotmail.com) upon reasonable request andsubject to approval by the Ethics Committee of the Hospital Universitario Ramón y Cajal.
