# Supplementary material for: Biomarker-defined congestion profiles in very old adults with acute heart failure: beyond ejection fraction classification
Source: Eur Geriatr Med. 2026 May 25;17(4):2073–85. doi: 10.1007/s41999-026-01505-y (PMC13562125; doi:10.1007/s41999-026-01505-y)
Supplement: Supplementary file 1 [file 41999_2026_1505_MOESM1_ESM.docx]

**Table S1. Multivariable Cox regression analysis for heart failure rehospitalization**

| **Variable** | **Hazard Ratio (HR)** | **95% CI** | **p value** |
| --- | --- | --- | --- |
| **Clinical and demographic covariates** | | | |
| Age (per year) | 1.02 | 1.00–1.04 | 0.092 |
| Female sex | 0.86 | 0.66–1.13 | 0.289 |
| Systolic BP (per mmHg) | 1.00 | 1.00–1.00 | 0.169 |
| Creatinine (per mg/dL) | 1.00 | 0.99–1.01 | 0.667 |
| Hemoglobin (per g/dL) | 1.03 | 0.97–1.09 | 0.361 |
| Pleural effusion | 1.13 | 0.87–1.46 | 0.356 |
| Peripheral edema | 1.05 | 0.80–1.38 | 0.702 |
| LVEF (per %) | 1.01 | 1.00–1.02 | 0.133 |
| **Biomarker-defined congestion phenotype (ref: C1)** | | | |
| C2 — High BNP / Low CA125 | 1.14 | 0.78–1.67 | 0.489 |
| C3 — Low BNP / High CA125 | 1.45 | 1.03–2.03 | 0.031 |
| C4 — High BNP / High CA125 | 1.31 | 0.93–1.83 | 0.123 |

Adjusted for age, sex, systolic blood pressure, creatinine, hemoglobin, pleural effusion, peripheral edema, and LVEF. Biomarker phenotypes: C1 (Low BNP/Low CA125, reference); C2 (High BNP/Low CA125); C3 (Low BNP/High CA125); C4 (High BNP/High CA125). BP, blood pressure; CI, confidence interval; HR, hazard ratio; LVEF, left ventricular ejection fraction.
