# Supplementary material for: Biomarker-defined congestion profiles in very old adults with acute heart failure: beyond ejection fraction classification
Source: Eur Geriatr Med. 2026 May 25;17(4):2073–85. doi: 10.1007/s41999-026-01505-y (PMC13562125; doi:10.1007/s41999-026-01505-y)
Supplement: Supplementary file 2 [file 41999_2026_1505_MOESM2_ESM.docx]

**Table S2. Multivariable Cox regression analyses with BNP and CA125 as continuous covariates**

**A. Outcome: All-cause mortality**

| **Variable** | **Hazard Ratio** | **95% CI** | **p value** |
| --- | --- | --- | --- |
| **Clinical and demographic covariates** | | | |
| Age (per year) | 1.053 | 1.033–1.074 | < 0.001 |
| Female sex | **0.731** | 0.578–0.925 | **0.009 *** |
| Systolic BP (per mmHg) | 1.001 | 0.998–1.003 | 0.563 |
| Creatinine (per mg/dL) | 0.998 | 0.991–1.006 | 0.631 |
| Hemoglobin (per g/dL) | 0.974 | 0.926–1.024 | 0.295 |
| Pleural effusion | 1.147 | 0.918–1.433 | 0.228 |
| Peripheral edema | 0.914 | 0.724–1.154 | 0.448 |
| LVEF (per %) | 1.004 | 0.994–1.013 | 0.455 |
| **Biomarkers (continuous)** | | | |
| **BNP (per pg/mL)** | **1.0003** | 1.0001–1.0006 | **0.001 *** |
| **CA125 (per U/mL)** | **1.0012** | 1.0003–1.0021 | **0.008 *** |
| **Interaction term** | | | |
| CA125 × BNP | ~1.00 | ~1.00–1.00 | 0.333 |
|  | | | |

** p < 0.05. Bold values indicate statistical significance. Adjusted for age, sex, systolic blood pressure, creatinine, hemoglobin, pleural effusion, peripheral edema, and LVEF. BNP and CA125 modelled as continuous variables; interaction term tested as c.CA125 × c.BNP. BP, blood pressure; BNP, B-type natriuretic peptide; CA125, cancer antigen 125; CI, confidence interval; HR, hazard ratio; LVEF, left ventricular ejection fraction.*

**B. Outcome: Heart failure rehospitalization**

| **Variable** | **Hazard Ratio** | **95% CI** | **p value** |
| --- | --- | --- | --- |
| **Clinical and demographic covariates** | | | |
| Age (per year) | 1.016 | 0.997–1.035 | 0.108 |
| Female sex | 0.851 | 0.649–1.115 | 0.242 |
| Systolic BP (per mmHg) | 1.001 | 0.999–1.004 | 0.159 |
| Creatinine (per mg/dL) | 0.998 | 0.989–1.007 | 0.677 |
| Hemoglobin (per g/dL) | 1.028 | 0.971–1.088 | 0.347 |
| Pleural effusion | 1.147 | 0.889–1.478 | 0.291 |
| Peripheral edema | 1.084 | 0.827–1.422 | 0.559 |
| **LVEF (per %)** | **1.013** | 1.002–1.025 | **0.018 *** |
| **Biomarkers (continuous)** | | | |
| **BNP (per pg/mL)** | **1.0003** | 1.0001–1.0006 | **0.019 *** |
| CA125 (per U/mL) | 1.0010 | 0.9998–1.0022 | 0.113 |
| **Interaction term** | | | |
| CA125 × BNP | ~1.00 | ~1.00–1.00 | 0.489 |
|  | | | |

** p < 0.05. Bold values indicate statistical significance. Adjusted for age, sex, systolic blood pressure, creatinine, hemoglobin, pleural effusion, peripheral edema, and LVEF. BNP and CA125 modelled as continuous variables; interaction term tested as c.CA125 × c.BNP. BP, blood pressure; BNP, B-type natriuretic peptide; CA125, cancer antigen 125; CI, confidence interval; HR, hazard ratio; LVEF, left ventricular ejection fraction.*
